# Dietary nitrate supplementation for preventing and reducing the severity of winter infections, including COVID-19, in care homes (BEET-Winter): a randomised placebo-controlled feasibility trial

**DOI:** 10.1007/s41999-022-00714-5

**Published:** 2022-11-16

**Authors:** Philip M. Bath, Cameron J. C. Skinner, Charlotte S. Bath, Lisa J. Woodhouse, Anastasia Areti Kyriazopoulou Korovesi, Hongjiang Long, Diane Havard, Christopher M. Coleman, Timothy J. England, Valerie Leyland, Wei Shen Lim, Alan A. Montgomery, Simon Royal, Amanda Avery, Andrew J. Webb, Adam L. Gordon, for BEET-Winter Investigators

**Affiliations:** 1 Stroke Trials Unit, Mental Health and Clinical Neuroscience, School of Medicine, University of Nottingham, South Block D Floor, Nottingham, NG7 2UH UK; 2 Stroke, Nottingham University Hospitals NHS Trust, Nottingham, NG7 2UH Nottinghamshire UK; 3 School of Biosciences, University of Nottingham, Sutton Bonington, LE12 5RD UK; 4 Division of Infection, Immunity and Microbes, School of Life Sciences, University of Nottingham, Nottingham, NG7 2UH UK; 5 Department of Stroke, University Hospitals of Derby and Burton, Derby, DE22 3NE UK; 6 Bramcote, Nottingham, NG9 UK; 7 Respiratory Medicine, Nottingham University Hospitals NHS Trust, Nottingham, NG5 1PB UK; 8 Nottingham Clinical Trials Unit, School of Medicine, University of Nottingham, Nottingham, NG7 2RD UK; 9 University of Nottingham Health Service, Cripps Health Centre, University Park, Nottingham, NG7 2QW UK; 10 Clinical Pharmacology, School of Cardiovascular Medicine and Sciences, Kings College London and British Heart Foundation Centre of Research Excellence, St Thomas’ Hospital, London, SE1 7EH UK; 11 Injury, Recovery and Inflammation Sciences, School of Medicine, University of Nottingham, Derby, DE22 3NE Derbyshire UK; 12 NIHR Applied Research Collaboration-East Midlands (ARC-EM), Nottingham, UK

**Keywords:** Care home, COVID-19, Dietary supplementation, Infection, Nitrate, Nitric oxide

## Abstract

**Purpose:**

Infections cause considerable care home morbidity and mortality. Nitric oxide (NO) has broad-spectrum anti-viral, bacterial and yeast activity in vitro. We assessed the feasibility of supplementing dietary nitrate (NO substrate) intake in care home residents.

**Methods:**

We performed a cluster-randomised placebo-controlled trial in UK residential and nursing care home residents and compared nitrate containing (400 mg) versus free (0 mg daily) beetroot juice given for 60 days. Outcomes comprised feasibility of recruitment, adherence, salivary and urinary nitrate, and ordinal infection/clinical events.

**Results:**

Of 30 targeted care homes in late 2020, 16 expressed interest and only 6 participated. 49 residents were recruited (median 8 [interquartile range 7–12] per home), mean (standard deviation) age 82 (8) years, with proxy consent 41 (84%), advance directive for hospital non-admission 8 (16%) and ≥ 1 doses of COVID-19 vaccine 37 (82%). Background dietary nitrate was < 30% of acceptable daily intake. 34 (76%) residents received > 50% of juice. Residents randomised to nitrate vs placebo had higher urinary nitrate levels, median 50 [18–175] v 18 [10–50] mg/L, difference 25 [0–90]. Data paucity precluded clinical between-group comparisons; the outcome distribution was as follows: no infection 32 (67%), uncomplicated infection 0, infection requiring healthcare support 11 (23%), all-cause hospitalisation 5 (10%), all-cause mortality 0. Urinary tract infections were most common.

**Conclusions:**

Recruiting UK care homes during the COVID-19 pandemic was partially successful. Supplemented dietary nitrate was tolerated and elevated urinary nitrate. Together, infections, hospitalisations and deaths occurred in 33% of residents over 60 days. A larger trial is now required.

**Trial Registration:**

ISRCTN51124684. Application date 7/12/2020; assignment date 13/1/2021.

**Supplementary Information:**

The online version contains supplementary material available at 10.1007/s41999-022-00714-5.

## Key summary points

**Aim:**

To assess the feasibility of supplementing dietary nitrate (NO substrate) intake in care home residents.

**Findings:**

Expressions of interest by care homes were not realised during the alpha COVID-19 wave of infections. Background dietary nitrate was < 30% of acceptable daily intake; most residents received a majority of their nitrate supplementation and supplementation increased urinary and salivary nitrate concentrations.

**Message:**

Recruiting UK care homes during the COVID-19 pandemic was partially successful and supplemented dietary nitrate was tolerated and elevated urinary nitrate concentrations.

**Supplementary Information:**

The online version contains supplementary material available at 10.1007/s41999-022-00714-5.

## Background

Epidemic winter infections due to viruses and bacteria cause considerable morbidity and mortality in care homes [1]. Common viral causes include influenza A/B viruses, parainfluenza virus, respiratory syncytial virus (RSV), rhinovirus and coronaviruses (CoVs: 229E, NL63, OC43, HKU1). Bacterial causes of respiratory infection include *Chlamydia pneumoniae*, *Haemophilus influenzae*, *Legionella* spp. and *Streptococcus pneumoniae* [1]. Care homes also have winter outbreaks of gastrointestinal tract infections (e.g. viral gastroenteritis due to norovirus), urinary tract, and skin and soft-tissue infections [1]. Overtaking all of these in 2020/21 was the SARS-CoV-2 pandemic which has had catastrophic consequences [2] with a third of excess deaths occurring in care homes and a reduction in resident life expectancy by 6 months [3]. Despite significant enhancements made to infection control procedures in care homes (hygiene, personal protective equipment) and prophylaxis with vaccination, infections have continued, and it is likely that event rates will return to baseline as practices normalise after the pandemic. Co-located older people in care homes are at high-risk for outbreaks of infectious diseases and yet there are no general antimicrobial measures that have demonstrated prophylactic efficacy against such outbreaks. For example, interventions such as probiotic capsules [4] have failed to demonstrate efficacy.

Nitric oxide (NO) possesses broad-spectrum antimicrobial activity with substantial in vitro and some in vivo data demonstrating anti-viral, bacterial, protozoal and fungi/yeast activity [5]. The antimicrobial effects of NO and derivative ions such as peroxynitrite are mediated by effects on RNA/DNA and protein conformation [5]. NO also improves organ blood supply and has pro-endothelial and anti-inflammatory and antithrombotic effects mediated through anti-leucocyte and anti-platelet activity [6], and these may also contribute to its antimicrobial effects. Antiviral and antibacterial activity has been demonstrated against many common causes of respiratory, gastrointestinal and soft-tissue infections; of relevance here, NO and phosphodiesterase 5-inhibitors (which maintain cyclic guanosine monophosphate levels, NO’s second messenger) have antiviral effects against influenza [7, 8] and coronaviruses such as SARS-CoV-2 [9, 10]. A recent human trial showed that a NO-nasal spray accelerated nasal SARS-CoV-2 viral clearance [11]. Endogenous NO is made from dietary amino acids (L-arginine and L-citrulline) and nitrate [5]. Phase II-equivalent clinical trials have supported anti-microbial effects of NO made from acidified nitrite on cutaneous viral and bacterial infections, dietary nitrate on oral bacteria, and NO gas against some respiratory viral infections [5]. Further, NO has been shown to improve exercise performance and cognition in older people [12], potential benefits of relevance to care home residents. Although some common infections have vaccines available (e.g. influenza, SARS-CoV-2), many do not yet (e.g. RSV) and vaccinations may need to be combined with chemoprophylaxis for effective prevention, especially in a population where immunosenescence is normal [13, 14]. So, NO substrates and donors may be particularly relevant due to their potential generic antimicrobial effects, especially since resistance against NO appears to be rare [5] in contrast to that occurring with many specific antimicrobial agents [1].

Care home residents have low nitrate and nitrite intake, in part because care home food may be low in nitrate content whilst food wastage (food left on plates) is high. We hypothesised that high infection rates in care homes might reflect low dietary nitrate intake and supplementation might reduce infections. The design and preliminary results of BEET-Winter have been published as a pre-print [15].

## Methods

### Aim, design and setting

BEET-Winter was a prospective cluster-randomised, placebo-controlled, blinded endpoint phase IIb trial and assessed pre-exposure prophylaxis; we tested the feasibility of supplementing dietary nitrate (given as beetroot juice) to care home residents, tolerability of beetroot juice, effect of dietary nitrate on salivary and urinary nitrate/nitrite concentrations, and safety and proof of concept. Further details are provided in the Supplementary Information and protocol on the trial website (https://stroke.nottingham.ac.uk/beet-winter/). The trial is registered ISRCTN51124684, application date 7/12/2020, assignment date 13/1/2021.

### Rationale

The proposed trial was premised on the following: (i) NO has broad spectrum antimicrobial affects [5] and so may reduce infections and their severity including death; (ii) Dietary sources of NO have a very low risk of harm; (iii) Care home diets are low in dietary nitrate; (iv) SARS-CoV-2, respiratory epidemic and norovirus infections in care homes increase during the autumn, winter and spring months; (v) The symptoms of many respiratory infections overlap so it is not possible to reliably distinguish clinically between the causative organism (e.g. influenza virus vs. RSV vs SARS-CoV-2) in the absence of multiplex testing, which is not routinely deployed; (vi) Co-infections caused by two or more pathogens of concern may interact in as yet undetermined ways; and (vii) Most COVID-19 trials have focussed on interventions that are unlikely to have beneficial effects on other microbial pathogens.

### Eligibility

UK care homes, with and without nursing or dual registered, were eligible for inclusion if they looked after older people, were rated at least ‘adequate’ by the English Care Quality Commission and had a minimum of 18 residents (Supplementary Information).

### Randomisation and masking

As a cluster-randomised trial, care homes (and not residents) were randomised dynamically by computer algorithm using minimisation to balance across important baseline characteristics: type (without nursing vs dual registered or with nursing alone), prior SARS-CoV-2 infection in wave 1 of the pandemic, and size (≤ 32 vs > 32 residents). Ten percent of randomisations were based on chance. Randomised homes were assigned in a 1:1 ratio to receive nitrate-containing juice and usual care vs nitrate-free juice (placebo) and usual care. Residents, care homes and trials staff were unaware of the assigned treatments.

### Interventions

Provision of nitric oxide (NO) was via 70 ml of nitrate-containing (400 mg) beetroot juice (Beet It Beetroot Juice Sport Shot—70 ml, James White Ltd, Ipswich UK), which is metabolised to NO in vivo, via the nitrate-nitrite-NO pathway [16] and was given once daily for 60 days. Placebo was given in the form of 70 ml of nitrate-free (0 mg) beetroot juice (placebo Beet It Beetroot Juice Sport Shot—70 ml, James White Ltd) given once daily for 60 days.

Both active and placebo juices have been used in multiple previous clinical trials and are identical in appearance, smell and taste [17]. Both juices contain folate, potassium, vitamin C, fibre and antioxidants but no allergens, and can be stored at room temperature with a shelf life > 1 year. The juices are palatable to many, but if found unpalatable, taste can be masked by dilution in other juices, e.g. orange or apple juice, or consumption through a straw.

### Outcome measures

Feasibility outcomes included the following: recruitment of care homes; recruitment of residents; adherence to the intervention (75% of residents take > 50%); ability to take juice; assessment of salivary and urinary nitrate concentrations (using Quantofix nitrate/nitrite, Camlab, Cambridge UK) at 60 days; ability to measure the ordinal outcome measure; assessment of incident infection rate using the ordinal outcome; and estimation of the intra-cluster correlation (ICC). Background dietary nitrate intake was assessed from care home menus and photographs of lunch before and after consumption (Supplementary Information).

The efficacy outcome was the most serious event occurring during treatment from an ordinal clinical scale comprising: (1) all-cause mortality, (2) all-cause hospitalisation, (3) infection with the resident remaining in the care home but needing healthcare support (e.g. from the general practitioner, 111 call, 999 call/paramedic), (4) infection with the resident remaining in the care home and not needing healthcare support, and (5) no infection, at 60 days after randomisation; a further analysis assessed this outcome at 90 days. Other outcomes included the components of the efficacy outcome, efficacy outcome in pre-specified subgroups, time to asymptomatic and symptomatic proven SARS-CoV-2 infection, time to first admission to hospital and its cause, time to death and its cause, frailty (clinical frailty scale, CFS) [18], disability (Barthel index), cognition (6-item test) and quality of life (EuroQoL, EQ-5D-5L; EuroQoL visual analogue scale, EQ-VAS). Safety outcomes comprised reported serious adverse events (SAEs) and adverse events (AEs) which exclude any events contributing to the clinical primary outcome, i.e., not involving an infection, requiring hospitalisation or leading to death; SAEs were defined as those requiring healthcare support (i.e., medically important) and AEs as those not requiring healthcare support.

### Dietary nitrate/nitrite intake

Dietary nitrate and nitrite intake were estimated from eight lunch menus and 69 photographs of lunch plates before and after consumption from four residents in one participating care home. These data were supplemented by internet-uploaded menus from three other UK care homes. Combining all the gathered information and with a consensus agreement between three researchers (AA, AKK, HJL), a representative 4-days’ full food and drinks menu, with serving sizes, was created for the care homes. Results were adjusted for average food wastage using data previously reported for a care home in the UK [19].

### Procedures

Trial documents including protocol and training materials are hosted on an open website (https://stroke.nottingham.ac.uk/beet-winter/). Care Home staff were trained by the research trial team and then approached eligible care home residents for electronic informed consent, or relatives if the resident lacked capacity. Capacity was assessed using the ‘3 question approach’, as used in the RIGHT-2 trial [20].

Following consent/proxy consent, care home staff submitted data online (REDCap, Vanderbilt University, Nashville USA) at days 0 (baseline), 14, 60 (end-of-treatment) and 90 (final follow-up), and at the time of any outcome event or serious adverse event.

### Statistical analysis

The statistical analysis plan was published on 9 August 2021 at https://stroke.nottingham.ac.uk/beet-winter/. A total of 360 residents from 30 homes with 12 residents per home (range 12–17) would be needed assuming alpha 0.05, power 0.80, intra-cluster correlation (ICC) 0.01 (with assumptions based on previous studies, Supplementary Information Table 1). Up to six additional care homes, each with between 4 and 20 participants, could be added in case some homes dropped-out, or if fewer than 12 residents were recruited at some homes.

**Table 1:** Baseline characteristics

	All	Nitrate	Placebo
*Care home characteristics*	6	4	2
Homes (%)			
Residential	2 (33.3)	2 (50.0)	0 (0.0)
Mixed	3 (50.0)	1 (25.0)	2 (100.0)
Nursing	1 (16.7)	1 (25.0)	0 (0.0)
Region (%)			
East Midlands	5 (83.3)	3 (75.0)	2 (100.0)
South-east	1 (16.7)	1 (33.3)	0 (0.0)
Last CQC rating (%)			
Excellent	2 (33.3)	1 (25.0)	1 (50.0)
Good	4 (66.7)	3 (75.0)	1 (50.0)
Needs improvement	0 (0.0)	0 (0.0)	0 (0.0)
Client age group (%)			
Older adults, 65 + years	5 (83.3)	3 (75.0)	2 (100.0)
Mixed, 18 + years	0 (0.0)	0 (0.0)	0 (0.0)
Younger adults, 18–65 years	1 (16.7)	1 (25.0)	0 (0.0)
Number of residents	68.0 (25.8)	68.0 (29.8)	68.0 (25.5)
Number of registered nurses	9.0 (9.6)	5.8 (7.2)	15.5 (13.4)
Number of non-nurse carers	84.8 (47.3)	61.3 (35.9)	132.0 (25.5)
Ratio residents to staff	1.2 (1.2)	1.6 (1.4)	0.5 (0.1)
Staff vaccinated against flu (%)	6 (100.0)	4 (100.0)	2 (100.0)
Taken part in previous research (%)	4 (66.7)	2 (50.0)	2 (100.0)
*Resident characteristics*			
Number	49	28	21
Age (years)	82.3 (8.2)	81.6 (8.5)	83.1 (7.9)
> = 70 (%)	31 (63.3)	15 (53.6)	16 (76.2)
Sex, female (%)	31 (63.3)	19 (67.9)	12 (57.1)
Race-ethnicity, non-white (%)	0 (0.0)	0 (0.0)	0 (0.0)
Care home (%)	8.0 [7.0, 12.0]	7.5 [4.0, 10.0]	10.5 [8.0, 13.0]
Residential	16 (32.7)	16 (57.1)	0 (0.0)
Nursing	33 (67.3)	12 (42.9)	21 (100.0)
Time in home (days) [IQR]	474.0 [284.0, 1053]	460.5 [303.0, 1073]	501.0 [274.0, 967.0]
Advance directive—no hospitalisation (%)	8 (16.3)	2 (7.1)	6 (28.6)
Do not attempt resuscitation order (%)	40 (81.6)	21 (75.0)	19 (90.5)
Lacked capacity (%)	41 (83.7)	22 (78.6)	19 (90.5)
Medical history (%)			
Blood, e.g. lymphoma, myeloma	2 (4.4)	1 (3.6)	1 (5.9)
Brain, e.g. PD, MS	10 (22.2)	7 (25.0)	3 (17.6)
Cancer, under therapy	6 (13.3)	4 (14.3)	2 (11.8)
COVID-19	9 (20.0)	4 (14.3)	5 (29.4)
Diabetes mellitus	2 (4.4)	1 (3.6)	1 (5.9)
Dementia	38 (84.4)	23 (82.1)	15 (88.2)
Headache/migraine	0 (0.0)	0 (0.0)	0 (0.0)
Heart, e.g. heart failure	14 (31.1)	8 (28.6)	6 (35.3)
Heart attack	4 (8.9)	2 (7.1)	2 (11.8)
Hypertension, on tablets	16 (35.6)	13 (46.4)	3 (17.6)
Hyperlipidaemia, on tablets	6 (13.3)	5 (17.9)	1 (5.9)
Kidney disease, chronic	5 (11.1)	4 (14.3)	1 (5.9)
Kidney stones	0 (0.0)	0 (0.0)	0 (0.0)
Leg ulceration, current	2 (4.4)	1 (3.6)	1 (5.9)
Liver, e.g. hepatitis, cirrhosis	0 (0.0)	0 (0.0)	0 (0.0)
Lung, e.g. asthma, COPD	6 (13.3)	4 (14.3)	2 (11.8)
Pneumonia	2 (4.4)	2 (7.1)	0 (0.0)
Stroke	8 (17.8)	4 (14.3)	4 (23.5)
Urinary catheter, current	3 (6.7)	2 (7.1)	1 (5.9)
Urinary tract infection	15 (33.3)	15 (53.6)	0 (0.0)
Interventions (%)			
Steroid tablets, current	0 (0.0)	0 (0.0)	0 (0.0)
Vitamin D supplementation	16 (34.8)	13 (46.4)	3 (16.7)
Weight (kg)	67.9 (15.6)	69.6 (18.1)	64.8 (9.8)
Height (m)	1.6 (0.1)	1.6 (0.1)	1.7 (0.1)
Body mass index (kg.m^−2^)^†^	25.3 (5.1)	26.3 (5.8)	23.5 (2.8)
Number of risk factors (/11)^†‡^	3.0 [3.0, 4.0]	3.5 [2.5, 4.0]	3.0 [3.0, 3.0]
*Clinical*			
Clinical frailty scale (/9)	7.0 [6.0, 7.0]	7.0 [6.0, 7.0]	6.0 [6.0, 7.0]
Barthel index (/100)	40.4 (29.4)	40.9 (28.5)	39.8 (31.2)
6 item cognition (/28)	28.0 [19.0, 28.0]	28.0 [18.5, 28.0]	28.0 [21.0, 28.0]
Quality of life, EQ-5D-5l (/1)	0.5 (0.2)	0.5 (0.2)	0.5 (0.2)
Quality-of-life, EQ-VAS (/100)	64.8 (18.2)	57.3 (15.9)	74.9 (16.3)

Data are number (%), median [interquartile range] or mean (standard deviation)

^†^Calculated

^‡^Sum of (age>70) + Blood + Brain + Cancer + Diabetes + Heart + Kidney + Liver + Lung + Steroid + (BMI>40)

Care home and resident characteristics and feasibility data are tabulated as number (%), median [interquartile range] or mean (standard deviation). Comparative analyses were to use a multi-level ordinal logistic regression model with adjustment for the minimisation factors and individual-level covariates (age, sex) and a random effect to adjust for clustering within care homes. The treatment comparison was to be presented as an adjusted common odds ratio (with 95% confidence intervals) for a shift in the direction of a better outcome on the ordinal scale [21]. Prespecified analyses of the efficacy outcome were to be performed in subgroups defined by the following adjustment factors: care home type (with/without nursing), prior SARS-CoV-2 infection in the care home, number of residents in care home, age, sex, vaccination status. Other outcomes were to be analysed using regression models dependent on data type (binary, categorical, continuous, time to event), adjusted similarly and accounting for clustering within care homes. All P values are two-sided and shown without adjustment for multiple testing. Analyses were performed using SAS version 9.4.

## Results

### Recruitment of care homes

**Fig. 1:**
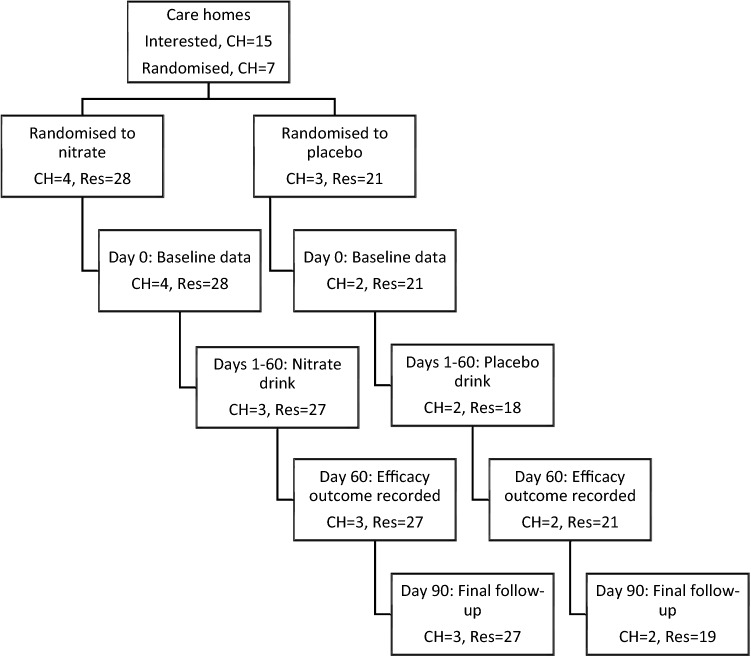
Flow chart. *CH*: care homes; Res: residents

Of an intended recruitment of 30 care homes, 16 expressed interest, 12 signed a contract, 7 received training, 6 consented residents and 5 treated and followed-up residents (Supplementary Information Tables 2 and 3, Fig. 1). Ethics submission was made via the UK Integrated Research application System (IRAS) system in mid-September 2020, i.e. prior to the start of the UK’s second COVID-19 wave (which was due to the wildtype/Wuhan variant) (Supplementary Information Table 4). However, the trial was subsequently rejected for urgent public health badging and so ethics review was not expedited; as a result, ethics approval was received in late November 2020 at the start of the UK’s third COVID-19 wave (due to alpha variant). Following contracting and training, the first care home was randomised in mid-December 2020 and started treatment in mid-January 2021. The trial timings are summarised in Supplementary Information Table 4. The median intervals between contact, contracting, juice testing, care home randomisation, training, juice arrival, consent, baseline assessment, start of juice and days 14, 60 and 90 are shown in Supplementary Information Table 5. Of the participating care homes, four were mixed or nursing and two had a CQC rating of excellent (Table 1).

### Recruitment of residents

A total of 49 residents were recruited from six care homes, median 8.0 [7.0, 12.0] per home. Baseline characteristics were mostly balanced between the groups and were largely representative of UK care home residents in respect of mean (SD) age 82 (8) years, sex with 63% female and no non-whites (Table 1). A majority of residents (84%) lacked capacity to consent and needed consultee consent and 16% had an advance directive for non-admission to hospital. Residents mostly had the following: dementia (84%), maximal cognitive impairment (six-item test 28 of 28), frailty (CFS 7 of 9), dependency (BI 40/100) and poor quality of life (health status 0.5/1.0, visual analogue scale 65/100) (Table 1). Two care homes (with 6 participating residents) withdrew altogether from the trial following randomisation and prior to collection of baseline data or commencement of beetroot juice.

### Adherence to beetroot juice

All 45 residents with data received at least one beetroot juice shot and 76% of residents took > 50% of drinks. The median [interquartile range] and (range) of drinks taken was, overall—40 [31, 47] (5–60), nitrate—46 [33, 58] (8–60) and placebo—32 [27, 43] (5–52).

### Salivary/urinary nitrate concentration

Five of the six care homes reported at least some nitrate measurements. Baseline urinary nitrate concentration was 10 [10, 50] mg/L. Salivary measurements were not available from the two homes randomised to placebo; in those randomised to nitrate, the median [interquartile range] salivary nitrate was 50 [50, 100] mg/L and median [interquartile range] salivary nitrite [10, 20] mg/L (Supplementary Information Table 7). An on-treatment analysis based on randomised clusters was not possible due to the paucity of participating care homes. However, since dietary nitrate intake was low (Supplementary Information Table 7), salivary and urinary levels will largely have depended on randomised intake and so a comparison of residents on active versus placebo juice was performed without accounting for clustering. In before–after (baseline/day 60) analyses, those randomised to nitrate-containing beetroot juice had non-significantly higher urinary nitrate at 60 days, change 15 [0, 75] mg/L (*p* = 0.12); by contrast, levels did not change in those randomised to placebo juice, difference 9.5 [−10 to 40] (*p* = 0.33). When adjusted for baseline, urinary nitrate was higher with nitrate than no nitrate by 25 [0, 90] mg/L (*p* = 0.008), a more than doubling in concentration in the treatment group (Supplementary Information Table 7).

### Clinical outcomes

Although we had originally intended to perform statistical comparisons between the randomised groups with adjustment for clustering on the basis that we had recruited 30 care homes, this plan was dropped when only six care homes followed up residents. As a result, the ICC was not estimated. Hence, we provide only descriptive results for the primary and secondary outcomes (Table 2). The ordinal primary outcome was measured in 48 of the 49 participating residents; a resident in a care home that only recruited one participant never received treatment or had follow-up performed. Following randomisation, no residents died during the 60 days of treatment, five (10%) were admitted to hospital, 11 (23%) had an infection with the resident remaining in the care home but needing healthcare support, none had an infection with the resident remaining in the care home and needing no healthcare support, and 32 (67%) had no infection (Table 2). A majority of infections were from the urinary tract with respiratory and cutaneous sources also present; no cases of COVID-19 or influenza were recorded. Causes for hospitalisation included infection, myocardial infarction, fall, need for thickener for dysphagia and having swallowed dentures. Secondary clinical outcomes were recorded in most residents at 60 days (Table 2).

**Table 2:** Clinical outcomes

	All	Nitrate	Placebo
Number of Residents	48	27	21
*Efficacy outcome*	48	27	21
Worst event	–	–	–
No event	32 (66.7)	14 (51.9)	18 (85.7)
Infection	0 (0.0)	0 (0.0)	0 (0.0)
Infection healthcare advice	11 (22.9)	10 (37.0)	1 (4.8)
Hospitalised, all cause	5 (10.4)	3 (11.1)	2 (9.5)
Died, all cause	0 (0.0)	0 (0.0)	0 (0.0)
*Secondary outcomes*			
First event	–	–	–
No event	32 (66.7)	14 (51.9)	18 (85.7)
Infection	0 (0.0)	0 (0.0)	0 (0.0)
Infection healthcare advice	12 (25.0)	11 (40.7)	1 (4.8)
Hospitalised, all cause	4 (8.3)	2 (7.4)	2 (9.5)
Died, all cause	0 (0.0)	0 (0.0)	0 (0.0)
First hospitalisation or death, time to	5 (10.4)	3 (11.1)	2 (9.5)
First infection, time to	14 (29.2)	13 (48.1)	1 (4.8)
Respiratory tract	1 (2.1)	1 (3.7)	0 (0.0)
Influenza†	0 (0.0)	0 (0.0)	0 (0.0)
COVID–19†	0 (0.0)	0 (0.0)	0 (0.0)
Norovirus†	0 (0.0)	0 (0.0)	0 (0.0)
Urinary tract†	11 (22.9)	10 (37.0)	1 (4.8)
Cutaneous†	1 (2.1)	1 (3.7)	0 (0.0)
Other	1 (2.1)	1 (3.7)	0 (0.0)
Number of infections	0.0 [0.0, 1.0] (*n* = 48)	0.0 [0.0, 1.0] (*n* = 27)	0.0 [0.0, 0.0] (*n* = 21)
Disposition (%)			
Care home	44 (97.8)	26 (96.3)	18 (100.0)
Home alone or with relative/friend	1 (2.2)	1 (3.7)	0 (0.0)
At another home	0 (0.0)	0 (0.0)	0 (0.0)
In hospital	0 (0.0)	0 (0.0)	0 (0.0)
Died	0 (0.0)	0 (0.0)	0 (0.0)
*Clinical scores*			
Clinical frailty scale (/9)	7.0 [5.5, 7.0] (*n* = 44)	7.0 [6.0, 7.0] (*n* = 27)	6.0 [4.0, 7.0] (*n* = 17)
Barthel index (/100)	40.0 (29.0) (*n* = 44)	40.9 (29.8) (*n* = 27)	38.5 (28.4) (*n* = 17)
6 item cognitive impairment (/28)	28.0 [20.5, 28.0] (*n* = 44)	28.0 [23.0, 28.0] (*n* = 27)	28.0 [19.0, 28.0] (*n* = 17)
Quality of life, EQ–5D–5L HSUV (/1)	0.5 (0.3) (*n* = 44)	0.5 (0.3) (*n* = 27)	0.6 (0.3) (*n* = 17)
Quality of life, EQ–VAS (/100)	60.5 (15.4) (*n* = 44)	58.3 (17.0) (*n* = 27)	63.9 (12.1) (*n* = 17)
Tolerability, > 70% of shots	19 (42.2)	14 (51.9)	5 (27.8)

Data are number (%), median (interquartile range] or mean (standard deviation)

^†^Estimated from symptoms

Serious adverse events (SAEs) and adverse events were diverse in nature and were, with the exception of one AE, only reported by care homes randomised to nitrate (Table 3).

**Table 3:** Serious adverse events and adverse events, by type

	Events		Residents	
	Nitrate	Placebo	Nitrate *N* = 27	Placebo *N* = 21
SAEs				
Agitation	2 (11.8)	0 (0.0)	2 (7.4)	0 (0.0)
Blocked ears	1 (5.9)	0 (0.0)	1 (3.7)	0 (0.0)
Confusion	1 (5.9)	0 (0.0)	1 (3.7)	0 (0.0)
Constipation	1 (5.9)	0 (0.0)	1 (3.7)	0 (0.0)
Dementia	1 (5.9)	0 (0.0)	1 (3.7)	0 (0.0)
Fall	2 (11.8)	0 (0.0)	1 (3.7)	0 (0.0)
Fluid retention	1 (5.9)	0 (0.0)	1 (3.7)	0 (0.0)
Hallucinations	1 (5.9)	0 (0.0)	1 (3.7)	0 (0.0)
Hypertension	1 (5.9)	0 (0.0)	1 (3.7)	0 (0.0)
Medications increased	2 (11.8)	0 (0.0)	1 (3.7)	0 (0.0)
Medications lowered/stopped	3 (17.6)	0 (0.0)	2 (7.4)	0 (0.0)
Rash	1 (5.9)	0 (0.0)	1 (3.7)	0 (0.0)
Total	17	0	9 (33.3)	0 (0.0)
Adverse events				
Blister	1 (3.4)	0 (0.0)	1 (3.7)	0 (0.0)
Faecal impaction	1 (3.4)	0 (0.0)	1 (3.7)	0 (0.0)
Fall	8 (27.6)	0 (0.0)	6 (22.2)	0 (0.0)
Fluid retention	1 (3.4)	0 (0.0)	1 (3.7)	0 (0.0)
Put on thickener	0 (0.0)	1 (100.0)	0 (0.0)	1 (4.8)
Rash	1 (3.4)	0 (0.0)	1 (3.7)	0 (0.0)
Seizure	13 (44.8)	0 (0.0)	1 (3.7)	0 (0.0)
Skinless area	2 (6.9)	0 (0.0)	1 (3.7)	0 (0.0)
Urinary retention	1 (3.4)	0 (0.0)	1 (3.7)	0 (0.0)
Vacant episode	1 (3.4)	0 (0.0)	1 (3.7)	0 (0.0)
Total	29	1	11 (40.7)	1 (4.8)

Data are number (%)

When assessed by individual care homes, it appeared that most primary outcome events, SAEs and AEs were reported by one home (labelled A, Table 4) that had been randomised to nitrate juice; two other homes randomised to nitrate reported few events (labelled B, C). Few clinical events were reported by the two homes randomised to nitrate-free juice (labelled E and F).

**Table 4:** Capacity, outcomes and SAEs by each randomised care home

Randomised group	Nitrate				No nitrate		
Care home	*A*	*B*	*C*	*D*^†^	*E*	*F*^‡^	*G*^§^
Participating residents	8	12	7	1	13	8	5
Lacked capacity (%)	4 (50)	12 (100)	6 (86)	0 (0)	12 (92)	7 (88)	1 (20)
Primary outcome, day 60							
No event	1 (13)	8 (67)	5 (71)	–	11 (85)	7 (88)	–
Infection	0 (0)	0 (0)	0 (0)	–	0 (0)	0 (0)	–
Infection healthcare advice	5 (63)	3 (25)	2 (29)	–	1 (8)	0 (0)	–
Hospitalised, all cause	2 (25)	1 (8)	0 (0)	–	1 (8)	1 (13)	–
Died, all cause	0 (0)	0 (0)	0 (0)	–	0 (0)	0 (0)	–
Falls, number of residents (%)	3 (38)	3 (25)	0 (0)	–	0 (0)	1 (13)	–
Serious adverse events (healthcare input)	8 (100)	1 (8)	0 (0)	–	0 (0)	0 (0)	–
Adverse events (no healthcare input)	4 (50)	7 (58)	0 (0)	–	0 (0)	1 (13)	–

Data are number (%)

^†^Care home was randomised and collected baseline data, but no juice administered, and no outcomes collected since care home chain decided home should not be in study

^‡^1 resident died prior to completion of consent so excluded. One resident had consultee assent which was then withdrawn prior to baseline

^§^Manager left and replacement did not want care home to participate so no baseline or outcome data collected, or juice administered

### Dietary nitrate/nitrite intake

Taking account of food nitrate and nitrite content [22], nitrate content in drinking water and the World Health Organization nitrate acceptable daily intake (ADI) of 0.6 mmol/kg (3.7 mg/kg) body weight and nitrite of 0.0015 mmol/kg (0.07 mg/kg) [23–25], and assuming a 60 kg care home resident, mean dietary nitrate and nitrite intake were low at 29.8% and 10%, respectively (Table 2).

### Vaccinations

Most (82%) residents had received one vaccination at baseline, and this rose to 96% by end of follow-up at day 90. Further, a majority (26, 57%) of residents had received double vaccination by day 90 (Supplementary Information Table 6). Both Astra-Zeneca and Pfizer vaccines were used. Almost all (44, 96%) residents had received influenza vaccination prior to baseline.

### Trial challenges

Apart from lack of UPH badging and so delayed ethics approval, multiple other challenges occurred (Supplementary Information Table 8). Importantly, three care homes withdrew, one when the owner overruled the manager’s decision to participate and two when the manager left, and their replacement did not wish to participate. One of these care homes had already been randomised and received but not unpacked juice; for efficiency, the juice was passed onto the final recruited care home. At the care home level, two protocol violations occurred, with juice being started before consent in one and before baseline data had been collected in the other; these care homes were retrained on the key importance of consent and baseline data collection.

Four care home managers reported back on their experiences after trial end (Supplementary Information Table 9). Most felt it had been important to have taken part in the trial and that data collection volume was appropriate. All had diluted the beetroot juice in apple juice for reasons of palatability.

## Discussion

Our study was designed to test the feasibility of performing a trial of nitrate supplementation to prevent infections in care homes and assess whether there was any signal of efficacy. Unfortunately, the trial failed to recruit the target of 30 care homes since its start was severely delayed during the autumn of 2020 and interested care homes withdrew. Nevertheless, some care homes and residents were recruited, beetroot juice was tolerated, and outcomes were recorded. Nitrate-containing beetroot juice significantly increased urinary nitrate and both salivary nitrate and nitrite concentrations in before-after analyses and, for urinary nitrate, when compared with placebo. However, the paucity of data precluded statistical analyses of the main outcomes and so proof of concept could not be tested. A majority of infections were localised to the urinary tract and no cases of COVID-19 or influenza were recorded, presumably reflecting high vaccination rates against these viruses.

The UK’s Urgent Public Health badging system has been lauded since it prioritised and made possible trials and other studies considered to have a reasonable probability of assessing the epidemiology of SARS-CoV-2 and of developing new treatments and diagnostics for COVID-19; examples include the VIVALDI [26] and CONDOR [27] studies in care homes and RECOVERY [28] trial in hospitals. However, the UPH system has also been criticised for preventing other COVID-19 related studies through lack of badging and delaying or abandoning of non-COVID-19 related research. The implication for BEET-Winter of not achieving UPH badging was to delay ethics approval by 7 weeks from late summer and before the second/wild-type wave to during the UK’s alpha wave of COVID-19 at which time care homes were focussing on protecting and caring for residents and managing staff sickness. Unsurprisingly, although a sufficient number of care homes had originally expressed interest, we were left with only 20% of these who delivered the trial. Hence, as a feasibility trial, BEET-Winter demonstrated that it was challenging to recruit during the pandemic without UPH badging; this system should be revised in any future pandemic so as not to prevent or delay studies from running. Since care home residents were disproportionately affected by COVID-19, it is unfortunate that we could not test an easy to administer dietary-based and inexpensive potential broad-spectrum anti-microbial agent. However, the impediments to starting should not apply outside a pandemic and so we anticipate that a trial testing the wider effects of dietary nitrate supplementation should be feasible.

Assessment of dietary nitrate intake using standard food composition tables is challenging [29], whilst recording dietary intake in nursing home residents is particularly demanding [30]. Nevertheless, care home residents appear to have a low dietary nitrate/nitrite intake that is less than 30% of acceptable daily intake, in part because high nitrate-containing foods such as leafy vegetables [22] are not prominent in menus and in part because food waste is high with leftovers on plates (14–25%) [19]. Hence, it is unsurprising that nitrate supplementation with beetroot juice significantly increased both salivary nitrate/nitrite and urinary nitrate concentrations, as has been reported previously in younger adults [31, 32]; such increases are likely to reflect higher plasma nitrate and nitrite concentrations [33]. In a recent study, survivors of COVID-19 infection had lower serum nitrite levels than a control uninfected group [34]. As has been seen in numerous trials in volunteers and older people with diabetes and hypertension, care home residents were largely adherent to both nitrate-containing and nitrate-free beetroot juice provided it was diluted in apple juice.

A key feasibility criterion was to collect infection, hospitalisation and fatal events and a third of residents had one of these. The paucity of data prevented us from comparing rates between the randomised groups. Further, many of these events as well as SAEs and AEs were reported by one home. Although event rates will vary between sites in any trial by chance, future trials will need to ensure thorough training so that common definitions of events are used and thus the propensity for differential reporting is reduced. Nevertheless, this observation does not detract from the possibility that dietary nitrate supplementation was associated with more events, SAEs and AEs, a question for a future trial.

Some of the technical difficulties faced with this trial replicate recurrent challenges seen in care home research. There is no care home research infrastructure in the UK outside of the Enabling Research in Care Homes (EnRICH) network [35], which focusses mainly on raising awareness of research and enabling co-operation with the care home sector. Care home staff are not routinely trained in Good Clinical Practice, many are research-naïve, and staff are substantially stretched to provide routine day-to-day care, even without the imposition of research. We have demonstrated with previous trials [36], and through this feasibility study, that these difficulties can be overcome through careful trial design. However, the superimposition of the COVID-19 pandemic rendered the research climate in care homes even more challenging with staff shortages, increased staff turnover and an increased burden of infection control activities that left even less time for research. We can reasonably assert that the objectives of this study would have been easier to achieve outside the context of the pandemic.

Having a cluster rather than individual randomised trial has both advantages and disadvantages. Cluster designs are especially relevant to care home trials since they reduce the risk of bias due to contamination [36], facilitate recruitment, enable the management and delivery of the intervention, and ease identification of serious adverse events in comparison with individual randomisation. Further, they most reflect the manner that prophylactic interventions will be used in care homes, i.e. for most residents. We have completed two cluster randomised trials in care homes, FICH [37] and FinCH [36, 38] and so elected to use a cluster trial design. Nevertheless, cluster randomisation leads to much larger trials than those using simple randomisation. In retrospect, it can be argued that we should have used individual randomisation with a resulting smaller sample size whilst accepting the relative weaknesses of this study design in a closed care home environment.

The strengths of this trial are the double-blind placebo-controlled design and assessment of resident dietary nitrate intake, juice tolerability in older people and effects on salivary and urinary nitrate/nitrite concentrations. Although long-term care sectors differ between countries, the COVID-19 pandemic has affected them similarly. If an evaluation of our intervention proved it to be effective, it is likely that such findings would be generalisable.

However, there are several caveats. First, we under-recruited care homes and so residents; this precluded statistical analysis that might have supported proof of concept. Second, there may have been differential care home reporting of outcomes and SAEs. Use of central routine data collection would remove this potential source of bias. Third, most reported infections were urinary tract infection, a notoriously unreliable diagnosis to make without laboratory support. Use of a multiplex approach might improve diagnosis and identification of the pathogen; a similar approach would help identification of microbes causing respiratory tract infection. Last, the use of a cluster design meant that far more care homes and residents were needed than if randomisation had been at the level of individuals.

## Conclusions

In summary, BEET-Winter tested the hypothesis that enhancing dietary inorganic nitrate intake might reduce care home infection rates during the winter, including COVID-19 and influenza rates. The trial started later than planned and during an active phase of the COVID-19 pandemic so that too few care homes, and so residents, were recruited. As a result, we were not able to test proof of concept and will need to repeat this assessment. However, the low dietary nitrate intake seen in care homes and increase in salivary and urinary concentrations seen with supplementation mean that antimicrobial effects should be obvious if preclinical and phase II-equivalent evidence of the effect of NO donors on bacteria and viruses [5] translates clinically. Since NO donors have vasculoprotective effects, and potential benefits on cognition, a larger trial might include these in a combined outcome. Nevertheless, adequate training will need to be performed with care home staff to reduce differential reporting of outcomes and SAEs.

## Supplementary Information

Below is the link to the electronic supplementary material.

Supplementary file1 (DOCX 86 kb)

## Data Availability

The data are available from the corresponding author on reasonable request with a protocol including analysis plan.

## References

[1] Strausbaugh LJ, Sukumar SR, Joseph CL (2003). Infectious disease outbreaks in nursing homes: an unappreciated hazard for frail elderly persons. Clin Infect Dis.

[2] Comas-Herrera A, Zalakaín J, Litwin C, Hsu AT, Lemmon E, Henderson D, et al. (2020) Mortality associated with COVID-19 outbreaks in care homes: early international evidence. In: LTCcovidorg, International Long-Term Care Policy Network, CPEC-LSE.

[3] Burton JK, Reid M, Gribben C, Caldwell D, Clark DN, Hanlon P, et al. (2021) Impact of COVID-19 on care-home mortality and life expectancy in Scotland. medRxiv. 10.1101/2021.01.15.21249871 10.1093/ageing/afab080 PMC8135527 33914870

[4] Butler CC, Lau M, Gillespie D, Owen-Jones E, Lown M, Wootton M (2020). Effect of probiotic use on antibiotic administration among care home residents: a randomized clinical trial. JAMA.

[5] Bath PM, Coleman CM, Gordon AL, Lim WS, Webb AJ (2021) Nitric oxide for the prevention and treatment of viral, bacterial, protozoal and fungal infections. F1000Res 10: 536 10.12688/f1000research.51270.1 PMC9171293 35685687

[6] Mills CE, Khatri J, Maskell P, Odongerel C, Webb AJ (2017). It is rocket science—why dietary nitrate is hard to 'beet'! Part II: further mechanisms and therapeutic potential of the nitrate-nitrite-NO pathway. Br J Clin Pharmacol.

[7] Tonew E, Indulen MK, Dzeguze DR (1982). Antiviral action of dipyridamole and its derivatives against influenza virus A. Acta Virol.

[8] Rimmelzwaan GF, Baars MM, de Lijster P, Fouchier RA, Osterhaus AD (1999). Inhibition of influenza virus replication by nitric oxide. J Virol.

[9] Akaberi D, Krambrich J, Ling J, Luni C, Hedenstierna G, Järhult JD (2020). Mitigation of the replication of SARS-CoV-2 by nitric oxide in vitro. Redox Biol.

[10] Liu X, Li Z, Liu S, Sun J, Chen Z, Jiang M (2020). Potential therapeutic effects of dipyridamole in the severely ill patients with COVID-19. Acta Pharm Sin B.

[11] Tandon M, Wu W, Moore K, Winchester S, Tu YP, Miller C (2022). SARS-CoV-2 accelerated clearance using a novel nitric oxide nasal spray (NONS) treatment: a randomized trial. Lancet Reg Health Southeast Asia.

[12] Stanaway L, Rutherfurd-Markwick K, Page R, Ali A (2017) Performance and health benefits of dietary nitrate supplementation in older adults: a systematic review. Nutrients 9. 10.3390/nu9111171 PMC5707643 29077028

[13] Bradley SF (1999). Prevention of influenza in long-term-care facilities. Long-term-care committee of the society for healthcare epidemiology of America. Infect Control Hosp Epidemiol.

[14] Cox LS, Bellantuono I, Lord JM, Sapey E, Mannick JB, Partridge L (2020). Tackling immunosenescence to improve COVID-19 outcomes and vaccine response in older adults. Lancet Healthy Longev.

[15] Bath PM, Skinner CJC, Bath CS, Woodhouse LJ, Kyriazopoulou-Korovesi AA, Long HJ (2022). Dietary nitrate supplementation for preventing and reducing the severity of winter infections, including COVID-19, in care homes (BEET-Winter)—a randomised placebo-controlled feasibility trial. Res Square.

[16] Khatri J, Mills CE, Maskell P, Odongerel C, Webb AJ (2017). It is rocket science—why dietary nitrate is hard to ‘beet’! Part I: twists and turns in the realization of the nitrate-nitrite-NO pathway. Br J Clin Pharmacol.

[17] Mills CE, Govoni V, Faconti L, Casagrande ML, Morant SV, Crickmore H (2020). A randomised, factorial trial to reduce arterial stiffness independently of blood pressure: proof of concept? The VaSera trial testing dietary nitrate and spironolactone. Br J Clin Pharmacol.

[18] Rockwood K, Song X, MacKnight C, Bergman H, Hogan D, McDowell I (2005). A global clinical measure of fitness and frailty in elderly people. Can Med Assoc J.

[19] Cunneen S, Jones J, Davidson HIM, Bannerman E (2010). An investigation into food provision and consumption in a care home setting in the UK. Proc Nutr Soc.

[20] RIGHT-2 Investigators (2019) Prehospital transdermal glyceryl trinitrate in patients with ultra-acute presumed stroke (RIGHT-2): an ambulance-based, randomised, sham-controlled, blinded, phase 3 trial. Lancet 393:1009–1020. 10.1016/S0140-6736(19)30194-1 PMC6497986 30738649

[21] Bath PMW, Geeganage C, Gray LJ, Collier T, Pocock S (2008). Use of ordinal outcomes in vascular prevention trials: comparison with binary outcomes in published trials. Stroke.

[22] Hord NG, Tang Y, Bryan NS (2009). Food sources of nitrates and nitrites: the physiologic context for potential health benefits. Am J Clin Nutr.

[23] Katan MB (2009). Nitrate in foods: harmful or healthy?. Am J Clin Nutr.

[24] Lidder S, Webb AJ (2013). Vascular effects of dietary nitrate (as found in green leafy vegetables and beetroot) via the nitrate-nitrite-nitric oxide pathway. Br J Clin Pharmacol.

[25] Keller RM, Beaver L, Prater MC, Hord NG (2020). Dietary nitrate and nitrite concentrations in food patterns and dietary supplements. Nutr Today.

[26] Dutey-Magni PF, Williams H, Jhass A, Rait G, Lorencatto F, Hemingway H (2021). COVID-19 infection and attributable mortality in UK care homes: Cohort study using active surveillance and electronic records (March-June 2020). Age Ageing.

[27] Micocci M, Gordon AL, Seo MK, Allen AJ, Davies K, Lasserson D (2021). Is Point-of-Care testing feasible and safe in care homes in England? An exploratory usability and accuracy evaluation of a point-of-care polymerase chain reaction test for SARS-COV-2. Age Ageing.

[28] Horby P, Lim WS, Emberson JR, Mafham M, Bell JL, Linsell L (2020). Dexamethasone in Hospitalized Patients with Covid-19—preliminary report. N Engl J Med.

[29] Babateen AM, Fornelli G, Donini LM, Mathers JC, Siervo M (2018). Assessment of dietary nitrate intake in humans: a systematic review. Am J Clin Nutr.

[30] Buckinx F, Allepaerts S, Paquot N, Reginster JY, de Cock C, Petermans J (2017). Energy and nutrient content of food served and consumed by nursing home residents. J Nutr Health Aging.

[31] Bartholomew B, Hill MJ (1984). The pharmacology of dietary nitrate and the origin of urinary nitrate. Food Chem Toxicol.

[32] Pannala AS, Mani AR, Spencer JP, Skinner V, Bruckdorfer KR, Moore KP (2003). The effect of dietary nitrate on salivary, plasma, and urinary nitrate metabolism in humans. Free Radic Biol Med.

[33] Webb AJ, Patel N, Loukogeorgakis S, Okorie M, Aboud Z, Misra S (2008). Acute blood pressure lowering, vasoprotective, and antiplatelet properties of dietary nitrate via bioconversion to nitrite. Hypertension.

[34] Wang J, Mei F, Bai L, Zhou S, Liu D, Yao L (2021). Serum nitrite and nitrate: a potential biomarker for post-covid-19 complications?. Free Radic Biol Med.

[35] Davies SL, Goodman C, Manthorpe J, Smith A, Carrick N, Iliffe S (2014). Enabling research in care homes: an evaluation of a national network of research ready care homes. BMC Med Res Methodol.

[36] Robinson K, Allen F, Darby J, Fox C, Gordon AL, Horne JC (2020). Contamination in complex healthcare trials: the falls in care homes (FinCH) study experience. BMC Med Res Methodol.

[37] Walker GM, Armstrong S, Gordon AL, Gladman J, Robertson K, Ward M (2016). The falls in care home study: a feasibility randomized controlled trial of the use of a risk assessment and decision support tool to prevent falls in care homes. Clin Rehabil.

[38] Logan PA, Horne JC, Gladman JRF, Gordon AL, Sach T, Clark A (2021). Multifactorial falls prevention programme compared with usual care in UK care homes for older people: multicentre cluster randomised controlled trial with economic evaluation. BMJ.

