# Supplementary material for: Dietary nitrate supplementation for preventing and reducing the severity of winter infections, including COVID-19, in care homes (BEET-Winter): a randomised placebo-controlled feasibility trial
Source: Eur Geriatr Med. 2022 Nov 16;13(6):1343–55. doi: 10.1007/s41999-022-00714-5 (PMC9668238; doi:10.1007/s41999-022-00714-5)
Supplement: Supplementary file 1 — Supplementary file1 (DOCX 86 kb) [file 41999_2022_714_MOESM1_ESM.docx]

**SUPPLEMENTARY INFORMATION**

**Dietary nitrate supplementation for preventing and reducing the severity of winter infections, including COVID-19, in care homes (BEET-Winter)- a randomised placebo-controlled feasibility trial**

Philip M Bath, DSc FMedSci;^1,2^ Cameron J C Skinner, BSc MSc;^1^ Charlotte S Bath, BA;^1^ Lisa J Woodhouse, BSc MSc;^1^ Anastasia Areti Kyriazopoulou Korovesi, MSc;^3^ Hongjiang Long, MSc;^3^ Diane Havard, RN;^1^ Christopher M Coleman, PhD;^4^ Timothy J England, PhD FRCP;^1,5^ Valerie Leyland, MA;^6^ Wei Shen Lim, FRCP;^7^ Alan A Montgomery, PhD;^8^ Simon Royal, MBBS MRCGP;^9^ Amanda Avery, RD PhD;^3^ Andrew J Webb, PhD FRCP;^10^ Adam L Gordon, PhD FRCP;^11,12^ for BEET-WINTER Investigators

1. Stroke Trials Unit, Mental Health & Clinical Neuroscience, School of Medicine, University of Nottingham, Nottingham NG7 2UH UK
2. Stroke, Nottingham University Hospitals NHS Trust, Nottingham, Notts, NG7 2UH, UK
3. School of Biosciences, University of Nottingham, Sutton Bonington LE12 5RD UK
4. Division of Infection, Immunity and Microbes, School of Life Sciences, University of Nottingham, Nottingham NG7 2UH UK
5. Department of Stroke, University Hospitals of Derby and Burton, Derby DE22 3NE UK
6. Bramcote, Nottingham NG9 UK
7. Respiratory Medicine, Nottingham University Hospitals NHS Trust, Nottingham, NG5 1PB, UK
8. Nottingham Clinical Trials Unit, School of Medicine, University of Nottingham, Nottingham NG7 2RD UK
9. University of Nottingham Health Service, Cripps Health Centre, University Park, Nottingham NG7 2QW UK
10. Clinical Pharmacology, School of Cardiovascular Medicine & Sciences, Kings College London & British Heart Foundation Centre of Research Excellence, St Thomas' Hospital, London, SE1 7EH, UK
11. Injury, Recovery and Inflammation Sciences, School of Medicine, University of Nottingham, Derby, Derbyshire, DE22 3NE, UK
12. NIHR Applied Research Collaboration-East Midlands (ARC-EM), Nottingham UK

Correspondence to:

Prof Philip M Bath

Stroke Trials Unit, South Block D floor, Queen’s Medical Centre, Nottingham NG7 2UH

Tel: 0115 823 1765

Fax: 0115 823 1767

Email: [Philip.bath@nottingham.ac.uk](mailto:Philip.bath@nottingham.ac.uk)

ORCID: 0000-0003-2734-5132

**CONTENTS**

| **Content** | **Page** |
| --- | --- |
| Title | 1 |
| Participating care homes | 1 |
| Methods | 2 |
| Discussion | 3 |
| References | 4 |
| Supplementary Information Table 1 | 6 |
| Supplementary Information Table 2 | 7 |
| Supplementary Information Table 3 | 7 |
| Supplementary Information Table 4 | 8 |
| Supplementary Information Table 5 | 9 |
| Supplementary Information Table 6 | 10 |
| Supplementary Information Table 7 | 10 |
| Supplementary Information Table 8 | 11 |
| Supplementary Information Table 9 | 12 |
| Supplementary Information Table 10 | 13 |

The following Supplement provides further information on the trial design and additional results.

**PARTICIPATING CARE HOMES**

**Care homes who recruited and treated residents (number of residents)**

**Acer Court**: Nottingham, Nottinghamshire (1): Linda Simpson.

**Church farm Skylarks:** West Bridgford, Nottinghamshire (11): Rachel Williams, Samantha McCormack.

**Lynwood Court:** Ascot, Berkshire (7): Maxine Freeman, Bonnie Trevellyan, Vikki Ribeiro.

**Springbanks:** Chesterfield, Derbyshire (8): Karen Busby, Laura Hill.

**Wren Hall:** Selston, Nottinghamshire (12): Anita Astle, Sophie Martin, Damian Mann.

**Landermeads:** Chilwell, Nottinghamshire (13): Ros Heath, Kimberley Borton, Katy Jackson, Helen Rain.

**METHODS**

**Eligibility criteria**

**Care Home criteria**

***Inclusions***

- Ideally CQC good or outstanding rating

***Exclusions***

- Care homes where their staff slept in the care home at night
- Small homes <18

**Resident criteria**

***Inclusions***

- Age >=65
- Taking a normal / soft diet
- Willing to take treatment having taste-tested a beetroot shot

***Exclusions***:

- Participating in another randomised intervention trial
- No consent (resident, or family if resident lacks capacity)
- Using a thickener with food
- Feeding tube
- Using antiseptic mouthwash [1]
- Currently has an infection requiring hospitalisation
- Identified by care home staff to be in last few days of life
- Short-term respite care
- Care home staff
- Takes beetroot juice daily

**Discussion**

**Dietary nitrate/nitrite intake**

The acceptable daily intake (ADI) of nitrate was set by the World Health Organisation 60 years ago at 0.06 mmol/kg (3.7 mg/kg) equating to 4.3 mmol/day [260 mg/day] for a 70 kg adult.[2-4] Since care home residents have lower weights we adjusted this to 0.05 mmol [222 mg] for a 60 kg adult.

When assessing dietary nitrate intake, drinking water nitrate content should be included. Example dietary intake studies include one from 1989 where vegetarian diets contained ~4.3 mmol (260 mg0 nitrate/day, close to the ADI, and almost four times greater than a ‘normal’ diet, which contained ~1.2 mmol (73 mg) nitrate/day.[5] In a recent and larger study, vegetable diets graded in quintiles of daily nitrate intake contained: highest quintile median 2.3 mmol (141 mg), mid quintile median 1.0 mmol (59 mg), lowest quintile median 0.4 mmol (23 mg) per day.[6]

Our population of care home residents had estimated daily nitrate intake of <30% although this sits in the population range.[6]

**REFERENCES**

1. Bondonno, C.P., et al., *Antibacterial mouthwash blunts oral nitrate reduction and increases blood pressure in treated hypertensive men and women.* Am J Hypertens, 2015. **28**(5): p. 572-5.

2. Katan, M.B., *Nitrate in foods: harmful or healthy?* Am J Clin Nutr, 2009. **90**(1): p. 11-2.

3. Lidder, S. and A.J. Webb, *Vascular effects of dietary nitrate (as found in green leafy vegetables and beetroot) via the nitrate-nitrite-nitric oxide pathway.* Br J Clin Pharmacol, 2013. **75**(3): p. 677-96.

4. Keller, R.M., et al., *Dietary Nitrate and Nitrite Concentrations in Food Patterns and Dietary Supplements.* Nutrition Today, 2020. **55**(5): p. 218-226.

5. Taylor, S., *Relative Exposure to Nitrite, Nitrate, and N-NitrosoCompounds from Endogenous and Exogenous Sources.Food Toxicology*. 1989: Marcel Dekker Inc.

6. Bondonno, C.P., et al., *Vegetable nitrate intake, blood pressure and incident cardiovascular disease: Danish Diet, Cancer, and Health Study.* Eur J Epidemiol, 2021. **36**(8): p. 813-825.

7. Baldwin, N.S., et al., *Cluster randomised controlled trial of an infection control education and training intervention programme focusing on meticillin-resistant Staphylococcus aureus in nursing homes for older people.* J Hosp Infect, 2010. **76**(1): p. 36-41.

8. Chami, K., et al., *A short-term, multicomponent infection control program in nursing homes: a cluster randomized controlled trial.* J Am Med Dir Assoc, 2012. **13**(6): p. 569.e9-17.

9. Mody, L., et al., *A targeted infection prevention intervention in nursing home residents with indwelling devices: a randomized clinical trial.* JAMA Intern Med, 2015. **175**(5): p. 714-23.

10. Gravenstein, S., et al., *Comparative effectiveness of high-dose versus standard-dose influenza vaccination on numbers of US nursing home residents admitted to hospital: a cluster-randomised trial.* Lancet Respir Med, 2017. **5**(9): p. 738-746.

11. Gravenstein, S., et al., *Feasibility of a cluster-randomized influenza vaccination trial in U.S. nursing homes: Lessons learned.* Hum Vaccin Immunother, 2018. **14**(3): p. 736-743.

12. Loizeau, A.J., et al., *The trial to reduce antimicrobial use in nursing home residents with Alzheimer's disease and other dementias: study protocol for a cluster randomized controlled trial.* Trials, 2019. **20**(1): p. 594.

13. Arnold, S.H., et al., *Reducing Antibiotic Prescriptions for Urinary Tract Infection in Nursing Homes Using a Complex Tailored Intervention Targeting Nursing Home Staff: Protocol for a Cluster Randomized Controlled Trial.* JMIR Res Protoc, 2020. **9**(5): p. e17710.

14. Teesing, G.R., et al., *Improving Hand Hygiene Compliance in Nursing Homes: Protocol for a Cluster Randomized Controlled Trial (HANDSOME Study).* JMIR Res Protoc, 2020. **9**(5): p. e17419.

15. Sackley, C.M., et al., *An occupational therapy intervention for residents with stroke related disabilities in UK care homes (OTCH): cluster randomised controlled trial.* BMJ, 2015. **350**: p. h468.

16. Walker, G.M., et al., *The Falls In Care Home study: a feasibility randomized controlled trial of the use of a risk assessment and decision support tool to prevent falls in care homes.* Clin Rehabil, 2016. **30**(10): p. 972-983.

17. Logan, P.A., et al., *Multifactorial falls prevention programme compared with usual care in UK care homes for older people: multicentre cluster randomised controlled trial with economic evaluation.* BMJ, 2021. **375**: p. e066991.

**Supplementary Information Table 1**

**Design criteria in published cluster-randomised care home trials.**

|  | Target | N | Homes | N/home | Active | Control | RRR | ARR | ICC | Alpha | 1-ß |
| --- | --- | --- | --- | --- | --- | --- | --- | --- | --- | --- | --- |
| **Infection** |  |  |  |  |  |  |  |  |  |  |  |
| Baldwin 2010 [7] | MRSA | 480 | 24 | 20 | 15.3 | 17.0 | 10 | 1.7 | 0.01 | 5% | 80% |
| Chami 2012 [8] | All | 3,524 | 44 | 80 | 4.1 | 8.1 |  | 4.0 | 0.04 | 5% | 90% |
| Mody 2015 [9] | UTI | 418 | 12 | 35 |  |  | 23 S |  |  |  |  |
| Gravenstein 2017 [10] | Influenza | 75,917 | 823 | 92 | 3.4 | 3.9 | 11.2 | 0.5 | 0.35 | 5% | 80% |
| Gravenstein 2018 [11] | Influenza | 2,957 | 39 | 76 | 13.5 | 20.1 | 33.1 | 6.5 | ? | 5% | ? |
| Loizeau 2019 [12] | UTI/LRI | 410 | 28 | 15 |  |  |  | 0.38 | 0.01 | 5% | 90% |
| Arnold 2020 [13] | UTI | 1,274 | 22 | 58 | 0.30 | 0.15 | 50 | 0.15 | 0.07 | 5% | 80% |
| Teesing 2020 [14] | Hand hygiene |  | 45 | 6 | 50 | 35 |  | 15 | 0.40 | 5% | 80% |
| **Others, selected** |  |  |  |  |  |  |  |  |  |  |  |
| OTCH 2015 [15] | OT | 1042 | 228 | 4.6 | 5.5 | 5.3 |  | 0.2 | 0.37 | 5% | 90% |
| Walker 2016 [16] | Falls | 52 | 6 | 8.7 | 1.9 | 4.0 |  |  | - | - | - |
| Logan 2021 [17] | Falls | 1308 | 66 | 20 | 1.65pa | 2.5pa | 33% |  | 0.10 | 5% | 80% |

^ICC: intra-cluster correlation; LRI: lower respiratory tract infection; MRSA: methicillin-resistant^ *^Staphylococcus aureus^*^; OT: occupational therapy; UTI: urinary tract infection^

**Supplementary Information Table 2**

**Care home recruitment and status**

| Stage | No. of care homes |
| --- | --- |
| Intended | 30 |
| Expressions of interest | 16 |
| Contracts signed | 12 |
| Trained | 7 |
| Randomised | 7 |
| Recruited residents | 6 |
| Commenced juice | 5 |
| Completed juice | 5 |

**Supplementary Information Table 3**

**Feasibility outcomes**

| **Criteria** | **N (%)** |
| --- | --- |
| Recruitment of 30 homes | 7 (23) |
| Number of taste tests | 82 (21) |
| Recruitment of 384 residents | 49 (13) |
| Recruited / taste tested | 49 (60) |
| Assessment of salivary/urinary nitrate | 27 (55) |
| Ability to measure ordinal outcome, >90% | 48 (98) |
| Mortality, all cause | 0 (0) |
| Hospitalisation, all cause [10] | 5 (10) |
| Infection in care home, needed healthcare input | 11 (23) |
| Infection in care home, no healthcare input | 0 (0) |
| No infection | 32 (67) |
| Time to first infection [11] days, median [quartiles] | 22.5 [18.0, 56.0] |

**Supplementary Information Table 4**

**Timelines for trial**

| **Date** | **Event** |
| --- | --- |
| 28/07/20 | Protocol, first draft |
| 10/09/20 | UPH submission |
| 17/09/20 | IRAS submission |
| 21/09/20 | UPH rejection |
| 28/10/20 | Research Ethics Committee meeting (delay due to UPH rejection) |
| 09/11/20 | Research Ethics Committee, provisional opinion |
| 25/11/20 | Research Ethics Committee, final approval (ID 288542) |
| 26/11/20 | First contract with a care home |
| 07/12/20 | ISRCTN application |
| 21/12/20 | *Vaccinations at care homes commence in UK* |
| 16/12/20 | First randomisation of a care home |
| 22/12/20 | First care home received training on trial |
| 05/01/21 | First care home completes baseline form |
| 13/01/21 | ISRCTN assignment (delay due to COVID-19/Christmas) |
| 14/01/21 | First care home started nitrate supplementation |
| 05/06/21 | Last care home started nitrate supplementation |
| 14/03/21 | First care completed nitrate supplementation |
| 05/08/21 | Last care home completed nitrate supplementation |
| 06/10/21 | Last care home completed follow-up |

**Supplementary Information Table 5**

**Time to achieve milestones**

Data are days, median [25^th^, 75^th^ Quartiles]

|  | Contract | Testing | Random-isation | Training | Juice arrival | Consent | Baseline | Juice start | Day 14 | Day 60 | Day 90 |
| --- | --- | --- | --- | --- | --- | --- | --- | --- | --- | --- | --- |
| Contact | 141 [141, 141] | 161 [161, 229] | 182 [162, 231] | 200 [167, 224] | 186 [166, 239] | 235 [180, 251] | 235 [193, 251] | 248 [209, 269] | 265 [228, 284] | 309 [298, 335] | 356 [340, 356] |
| Contract | X | 21 [20, 80] | 42 [21, 82] | 60 [26, 60] | 46 [25, 86] | 87 [39, 95] | 87 [52, 95] | 108 [68, 128] | 125 [87, 143] | 169 [157, 194] | 215 [200, 215] |
| Testing |  | X | 2 [2, 8] | 14 [5, 16] | 10 [6, 12] | 27 [15, 28] | 31 [27, 32] | 48 [40, 56] | 67 [55, 71] | 137 [106, 148] | 179 [127, 187] |
| Random-isation |  |  | X | 6 [4, 14] | 4, [4, 4] | 19 [14, 26] | 29 [19, 31] | 47 [38, 54] | 66 [53, 69] | 127 [104, 136] | 158 [125, 185] |
| Training |  |  |  | X | -2 [-6, 0] | 13 [12, 27] | 27 [15, 27] | 43 [24, 48] | 62 [39, 65] | 109 [90, 132] | 140 [112, 190] |
| Juice arrival |  |  |  |  | X | 15 [10, 18] | 21 [15, 27] | 43 [30, 50] | 62 [45, 65] | 123 [96, 132] | 154 [117, 181] |
| Consent |  |  |  |  |  | X | 0 [0, 3] | 13 [12, 33] | 30 [27, 52] | 78 [74, 122] | 105 [99, 180] |
| Baseline |  |  |  |  |  |  | X | 13 [9, 16] | 30 [24, 35] | 75 [74, 105] | 105 [99, 163] |
| Juice start |  |  |  |  |  |  |  | X | 15 [15, 17] | 66 [63, 89] | 92 [92, 131] |
| Day 14 |  |  |  |  |  |  |  |  | X | 51 [48, 70] | 77 [75, 116] |
| Day 60 |  |  |  |  |  |  |  |  |  | X | 31 [29, 39] |
| Day 90 |  |  |  |  |  |  |  |  |  |  | X |

**Supplementary Information Table 6**

**Vaccination status against SARS-CoV-2**

Data are number (%). No Moderna vaccination was administered.

| **Vaccination** | **Day 0** | **Day 60** | **Day 90** |
| --- | --- | --- | --- |
| ***COVID-19 (%)*** |  |  |  |
| Single vaccination | 37 (82.2) | 38 (84.4) | 45 (95.7) |
| Pfizer | 18 (48.6) | 19 (50.0) | 19 (42.2) |
| AstraZeneca | 19 (51.4) | 19 (50.0) | 26 (57.8) |
| Time from first vaccination (days) | -75.0 [-81.0, -59.0] | -74.5 [-81.0, -20.0] | -67.0 [-81.0, 2.0] |
| Double vaccination | 0 (0.0) | 23 (53.5) | 26 (56.5) |
| Pfizer | 0 (0.0) | 17 (73.9) | 17 (65.4) |
| AstraZeneca | 0 (0.0) | 6 (26.1) | 9 (34.6) |
| Time from second vaccination (days) | - | 18.0 [-3.0, 74.0] | 18.0 [-3.0, 79.0] |
| ***Influenza (%)*** | 44 (95.7) | 27 (96.4) | 17 (94.4) |

**Supplementary Information Table 7**

**Salivary nitrate and nitrite, and urinary nitrate at baseline and whilst taking beetroot juice**

Data are mean (standard deviation); comparison by ANCOVA including adjustment for baseline concentration. Analyses do not take account of cluster randomisation.

| **Concent-ration** |  | **Baseline** |  | **On juice** |  | **Difference**  **(95% CI)** | **P** |
| --- | --- | --- | --- | --- | --- | --- | --- |
|  | **All** | **Nitrate** | **Placebo** | **Nitrate** | **Placebo** |  |  |
| Nitrate, urine | 47.1 (76.4) [n=27] | 52.2 (75.6) [n=18] | 36.8 (81.5) [n=9] | 117.0 (154.6) [n=20] | 43.0 (75.0) [n=10] | 74.0 (-32.5, 180.5) | 0.0097 |
| Nitrate, saliva (mg/L) | 91.1 (93.9) [n=9] | 91.1 (93.9) [n=9] | - | 247.2 (240.9) [n=9] | - | - | - |
| Nitrite, saliva (mg/L) | 15.8 (14.8) [n=9] | 15.8 (14.8) [n=9] | - | 40.1 (38.2) [n=9] | - | - | - |

**Supplementary Information Table 8**

**Issues with trial**

| **Issue** | **Implication** | **Remedy** |
| --- | --- | --- |
| ***Delay*** |  |  |
| Trial did not receive UPH badging | Delayed ethics approval and so start of trial and loss of multiple care homes. | None |
| Less interest by care homes in joining trial during second/third UK COVID-19 waves | Intended 30+ care homes not recruited | None |
| ***Staffing*** |  |  |
| High care home work-load exacerbated by COVID (PPE, increased testing, relative visits) | Reluctance to take part in research | Set research and network up before pandemic |
| ***Care home withdrawal*** |  |  |
| Commercial owner of CH overruled manager’s decision to participate | CH withdrawal in spite of having signed a contract to participate. This caused over-ordering of nitrate juice and unnecessary costs. | Contract with care home head-quarters prior to contacting individual care homes. |
| Manager left and new manager did not want to participate | CH withdrawal in spite of having signed a contract to participate. This caused over-ordering of nitrate juice and unnecessary costs. | None |
| ***Protocol violations*** |  |  |
| Juice started before consent | Protocol violation | Juice stopped. Retraining. |
| Juice started before baseline data collected | Protocol violation. Juice might have altered baseline data | Baseline data entered urgently. |
| **Event/SAE recording** |  |  |
| 2 sites entered far more events/SAEs than the other 4 sites | Both sites were randomised to nitrate, so event rate appears much higher in this group | Detected after data lock. Further training, more sites and individual randomisation would reduce this problem |
| ***Others*** |  |  |
| CH staff members did not have own work-related email address | Could not log into database | CH asked staff to use personal email addresses or share emails |

**Supplementary Information Table 9**

**Feedback from care home managers.**

| **Item** | **Median [IQR] / N (%)** |
| --- | --- |
| Number of residents | 52.0 [37.5, 59.5] |
| SARS-CoV-2 cases during Q1 2021 | 0.0 [0.0, 12.5] |
| Research in care homes |  |
| Appropriate to do research in this vulnerable population | 3 (75.0) |
| Research in care homes is beneficial | 4 (100.0) |
| COVID-19 research in care homes is timely | 3 (75.0) |
| I am happy that my care home was involved in this research | 2 (50.0) |
| Important that my care home took part | 3 (75.0) |
| Taking part will be useful in marketing the trial | 3 (75.0) |
| Recruitment of residents |  |
| Fewer than expected | 2 (50.0) |
| About what was expected | 2 (50.0) |
| More than expected | 0 (0.0) |
| Data collection |  |
| Far too little collected | 1 (25.0) |
| Too little collected | 0 (0.0) |
| About right collected | 2 (50.0) |
| Too much collected | 1 (25.0) |
| Far too much collected | 0 (0.0) |
| Data entry by |  |
| One staff member | 4 (100.0) |
| Two or more staff members | 0 (0.0) |
| Taste testing beetroot juice before enrolment was useful? | 4 (100.0) |
| Juice palatability |  |
| Drank neat | 0 (0.0) |
| Diluted with orange juice | 1 (25.0) |
| Diluted with apple juice | 4 (100.0) |
| Drunk through a straw | 0 (0.0) |
| Starting juice |  |
| All resident together | 3 (75.0) |
| Staggered start over several days | 1 (25.0) |
| Computers/tablets in care home |  |
| Enough | 3 (75.0) |
| Trial-dedicated tablet would be useful | 1 (25.0) |
| Guestimate on randomisation |  |
| Active | 1 (25.0) |
| Placebo | 2 (50.0) |
| Unknown | 1 (25.0) |
| ***Free text comments*** |  |
| Family feedback to manager | “interesting trial” |
| Care home manager | “completing data time-consuming” |

**Supplementary Information Table 10**

**Dietary nitrate and nitrite intake in care homes.**

Data are mean (standard deviation)

|  | ADI | Without water | With water |
| --- | --- | --- | --- |
| Nitrate, mg/day | 222 | 51.5 (44.4), 23.2% | 66.1 (56.9), 29.8% |
| Nitrite, mg/day | 4.2 | 0.4 (0.1), 10% | 0.4 (0.1), 10% |

ADI: acceptable daily intake, assuming 60 kg adult.[2-4]

Percentages represent proportion of ADI.
